# Inactivated *Lactobacillus* promotes protection against myocardial ischemia–reperfusion injury through NF-κB pathway

**DOI:** 10.1042/BSR20171025

**Published:** 2017-11-17

**Authors:** Ni Wang, Genhong Song, Yang Yang, Weiwei Yuan, Ming Qi

**Affiliations:** 1 Gerontology Department, Affiliated Hospital of Yan’an University (Shanxi), No. 43 North Street, Yan’an, Shanxi Province 716000, P.R. China; 2 Intensive Care Unit, Affiliated Hospital of Yan’an University (Shanxi), No. 43 North Street, Yan’an, Shanxi Province 716000, P.R. China; 3 Thoracis Surgery Department, Affiliated Hospital of Yan’an University (Shanxi), No. 43 North Street, Yan’an, Shanxi Province 716000, P.R. China

**Keywords:** inactivated lactobacillus, myocardial ischemia reperfusion injury, NF-κB, Oxygen free radical

## Abstract

Although restoration of blood flow to an ischemic organ is essential to prevent irreversible cellular injury, reperfusion may augment tissue injury in excess of that produced by ischemia alone. So this experiment was designed to study the protective effects and mechanism of inactivated *Lactobacillus* (Lac) on myocardial ischemia–reperfusion (I–R) injury (MIRI). MIRI rat models were established by ligation of left anterior descending coronary artery for ~30 min and then, reperfusion for 120 min and divided into control group, model group, and Lac (10^6^, 10^7^, and 10^8^ cfu/kg) groups. At the end of the test, the creatine kinase (CK) activity, lactate dehydrogenase (LDH) activity, superoxide dismutase (SOD) activity and malondialdehyde (MDA) content were assayed by corresponding kits. The heart was obtained from rats and the myocardial infarction area was determined by TTC staining and myocardial endothelial cell apoptosis rate was determined by Tunel kit. Besides, A20, IκB, nuclear factor (NF)-κB, and nitric oxide (NO) synthase (NOS) were also assayed by Western blot. When compared with model group, Lac obviously reduces MIRI in the rat by reducing myocardial infarction area and the apoptosis rate of endothelial cells; reduce the serum CK, LDH, and MDA content; increase the serum SOD activity; and suppress NF-κB signaling and NOS expression in the myocardial tissues. Lac pretreatment can inhibit lipid peroxidation and effectively improve MIRI caused by oxygen free radical through inhibiting NF-κB signaling.

## Introduction

Cellular damage after reperfusion of previously viable ischemic tissues is defined as ischemia–reperfusion (I–R) injury. Myocardial I–R injury (MIRI) refers to myocardial malfunction and damage after the reperfusion of previously viable ischemic cardiac tissues. Arrhythmia, expansion of infarction area, and reduced cardiac diastolic function usually occur after MIRI [1]. The mechanism of MIRI is complex including OFR/reactive oxygen species (ROS) role, calcium overload, and activation of neutrophils; however, oxygen free radicals (ROS) accumulation is known as one of the important mechanisms for MIRI [2]. Damage degree of MIRI decides the treatment and prognosis of ischemic heart disease. Currently, there is no breakthrough progress for prevention and control of MIRI.

Reperfusion of ischemic tissues results in the formation of toxic ROS, including superoxide anions (O^−^), hydroxyl radicals (OH^−2^), hypochlorous acid (HOCl), hydrogen peroxide (H_2_O_2_), and nitric oxide (NO) derived peroxynitrite. ROS are potent oxidizing and reducing agents which are produced by multiple sources during MIRI such as mitochondria, uncoupled eNOS, cytochrome P450, xanthine oxidase, and neutrophils [3–7]. Thus, an imbalance between excess production of free radicals during MIRI and inability of the body to counteract or detoxify their harmful effects through neutralization by antioxidants result into oxidative stress. As a consequence of oxidative stress, increased proinflammatory cytokine production and adhesion molecule expression, impaired endothelium-dependent vasodilation and increased endothelial permeability exacerbate myocardial damage and death. Oxidative stress can promote inflammation mainly through the activation of nuclear factor (NF)-κB [8].

Normal flora is widely distributed in the human body and owns important protective function against inflammation [9]. Previous study showed that inactivated *Pseudomonas aeruginosa* can effectively treat allergic inflammation, restore imbalance in immune system, and improve epithelial functions [10]. So, in the present study, we use an inactivated *Lactobacillus* (Lac) to observe its role in anti-inflammation, endothelial protection, and explore the protective effects of Lac against MIRI.

## Materials and methods

### Animal models

SD rats, half male and half female, weighing 200 ± 20 g were provided by Experimental Animal Center of Yan’an University (permit number: SXYA-2016 043). MIRI animal models were established by coronary artery ligation. The animals were fed a regular diet (crude protein: 19.31%, crude fat: 2.9%, crude fiber: 3.1%, moisture: 8.7%, calcium: 0.5%, and phosphorus: 0.5%). After preoperative fasting for 12 h, sodium pentobarbital of 35 mg/kg was injected intraperitoneally into rats for anesthesia and fixation. Then, the limbs were connected to ECG machine (1350 p-type with 12 leads). Then, trachea was separated and endotracheal intubation was inserted into the trachea, which was connected by animal rescue breathing machine (model: DW 3000; breathing rate: 60 times/min; tidal volume: 2 ml/100 g). Chest was opened along the rib gap between the third and fourth rib of left-sided sternum and the heart was exposed. The suture needle (needle depth: 0.1 cm) with line pass through the edge of left anterior descending coronary artery, putting a short diameter of 1.5 mm of latex tube after the line to tighten ligation to induce myocardial ischemia for ~30 min (the mark of success: ECG ST significantly up or T wave up). Then the ligation was loosened for reperfusion for ~120 min (the mark of a successful reperfusion: the elevated ST fell more than 50% or high T wave down). The animals with abnormal ECG or deaths before the completion of this procedure were excluded from the experiment.

### Grouping and drug delivery

Successful model rats were randomly divided into model group, Lac (L, 10^6^ cfu/kg) group, Lac (M, 10^7^ cfu/kg), and Lac (H, 10^8^cfu/kg), ten in each group. Another ten rats, sham-operation without coronary artery ligation, were used as a control group. Lac groups were treated intravenously daily for 3 days of preoperation and other two groups was given equal volume of 0.5% sodium CM-cellulose (CMC Na).

### Myocardial infarction area

TTC is the proton receptor of the mitochondrial respiratory chain pyridine nucleotide binding enzyme system, which can react with respiratory enzyme in normal tissues and exhibit red. However, respiratory enzyme activity decreased in ischemic tissues, which leads to no reaction between TTC and respiratory enzyme and appears pale. The myocardial infarction determined by TTC staining is 6 h earlier than that determined by the electron microscope and 24 h earlier than that by the light microscope. Rats were killed after taking blood, and the heart was obtained and rinsed in PBS buffer. The ventricular tissues were obtained and half of the tissues were cut into five pieces of equal thickness. The pieces were add into 2% TTC solution at 37°C for 10 min. Myocardial infarction was pale. With a digital camera image acquisition, image analysis software ImagePro Plus 7.0 was used for calculation of the ventricular infarction area and ventricular total area to obtain myocardial infarction rate (%).

### Apoptosis assay

The apoptosis was assayed by using Tunel apoptotic cells detection kit in strict guidance with the *in situ* apoptosis detection kit instructions. Anti-vwF (endothelial cell markers) antibody (1:200) was added to the slide at 4°C overnight in a wet box. Then the corresponding peroxidase-labeled secondary antibody (1:250) was added at 4°C for 1 h in dark room. DAB was used for coloration. Eight high visions (×200) were randomly selected from each section on ischemia and total number of cells and apoptosis cells were counted. Apoptotic index (AI) was calculated according to a percentage of apoptosis cell in the total endothelial cells.

### Determination of serum CK, LDH, LDH and MDA

After reperfusion, the femoral artery blood was extracted from animals of three groups and centrifuged at 3500 rev/min for 10 min to obtain serum. The creatine kinase (CK) and lactate dehydrogenase (LDH) activities were evaluated by colorimetric methods. The superoxide dismutase (SOD) activities were evaluated by hydroxylamine method while malondialdehyde (MDA) contents were evaluated by glucosinolates barbituric acid method, according to the kit’s instructions (Jiancheng, Nanjing, China).

### Western blot

The part of the ventricular tissues were washed with ice-cold PBS twice, respectively and resolved in RIPA buffer containing 1 mM PMSF, 5 mM β-glycerophosphate, and 1% of a standard protease inhibitor cocktail (Sigma Chemical Co.) on ice for 30 min. Insoluble materials were removed by centrifugation for 1 min at 10000×***g*** at 4°C. The supernatant was collected and the protein concentration was measured by Bradford method to be adjusted to a final concentration of 10 mg/ml. The supernatant were separated by SDS/PAGE and transferred on to nitrocellulose membrane. The membrane were blocked with 5% skim milk in PBS for 2 h and then incubated with polyclone rabbit anti-A20, anti-p-IκB, and anti-p65 antibody and appropriate horseradish peroxidase conjugated secondary antibody. Detection was done using the ECL system.

### Statistics

The software of SPSS 18.0 was used to analyze the results. The measurement data were expressed as mean ± S.D. (x ± s). Single factor ANOVA was used for comparison amongst multiple groups and *t* test was used for comparison between two groups. *P*<0.05 was recognized to be of statistical significance.

## Results

### Inactivated Lac reduces myocardial infarction area

**Figure 1:**
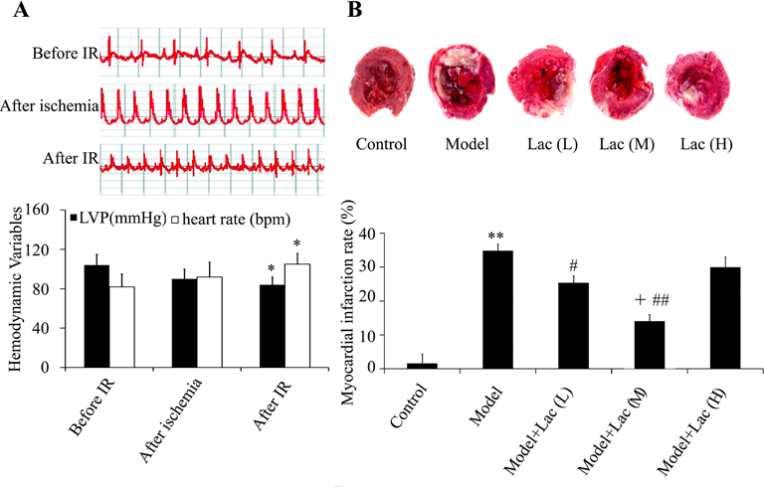
Cardiac function was assayed by ECG machine and TTC staining. (**A**) The ECG, LVP, and heart rates were recorded. (**B**) Myocardial infarction area was identified by TTC staining (*n*=5). The myocardial infarction area decreased in Lac groups. **P<0.01 compared with control; ^#^*P*<0.05, and ^##^*P*<0.01 compared with model group; ^+^*P*<0.05 compared with model + Lac (low) group * P<0.05 compared with before IR..

The results showed that after ischemia, the ST segment elevation was more than 0.1 mV or T waves ascended (Figure 1A) and the myocardial turned dark red. After 30 min, the ligation was released. The ST elevation decreased more than 1/2. The LVP increased and heart rates decreased after MIRI (*P*<0.05) (Figure 1A).

The results of TTC staining showed that the myocardial infarction area in model group was (34.51 ± 3.63)%, however, in inactivated Lac groups it was (22.17 ± 3.19)% (*P*<0.05) and (14.37 ± 1.98)% (*P*<0.01) at low concentration and medium concentration, respectively (Figure 1B), which indicated the obvious reduction in infarct area. Moreover, medium concentration of Lac showed the best protective effects.

### Inactivated Lac inhibits apoptosis of endothelial cells

**Figure 2:**
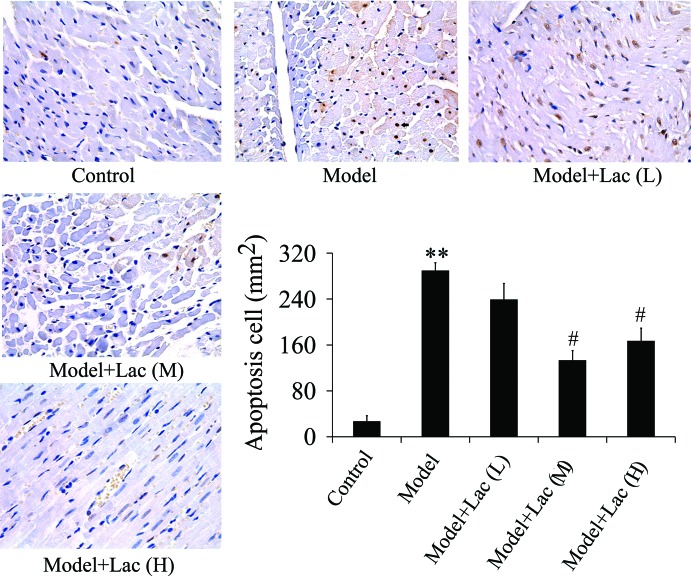
Apoptosis of endothelial cells were identified by TUNEL (*n*=4) The apoptosis of endothelial cells decreased significantly in Lac groups. **P<0.01 compared with control; ^#^*P*<0.05 compared with model group.

As shown in Figure 2, the apoptosis of endothelial cells in model group compared with the control group was significantly increased while in Lac (medium and high concentration) groups, it was decreased obviously. There were no significant differences between Lac (medium) and Lac (high) groups.

### Inactivated Lac reduces the activities of CK, LDH, MDA, and promotes the activity of SOD

**Figure 3:**
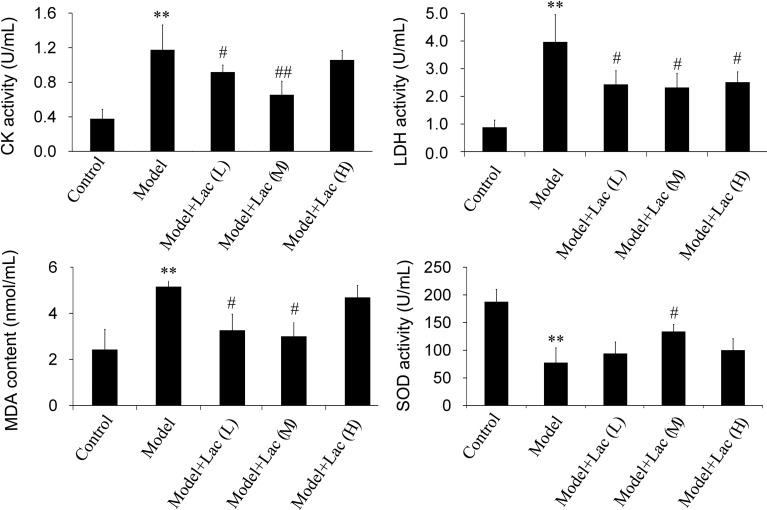
he activities of CK, LDH, MDA and SOD were assayed by biochemical kits (n=5). The activities of CK (**A**), LDH (**B**), MDA (**C**), and SOD (**D**) were assayed by biochemical kits (*n*=5). The serum activities of CK, LDH, and MDA increased obviously in model group, while Lac groups showed significant reduction in serum CK, LDH, and MDA activities. Moreover, in Lac (medium) group, the serum SOD activities were significantly increased. **P<0.01 compared with control; ^#^*P*<0.05 and ^##^*P*<0.01 compared with model group.

With 120-min reperfusion after 30-min myocardial ischemia, the serum activities of CK, LDH, and MDA increased obviously in model group (*P*<0.01) while inactivated Lac groups showed significant reduction in serum CK (Figure 3A), LDH (Figure 3B), and MDA (Figure 3C) activities. When compared with model group, the difference had statistical significance. However, there were no significant differences between Lac (low) and Lac (medium) groups. Besides, the serum SOD activity reduction was obvious (*P*<0.01) in model group while in inactivated Lac (medium) group, the serum SOD (Figure 3D) activities were significantly increased.

### Inactivated Lac increases the expression of A20 and decreases the activation of NF-κB

**Figure 4:**
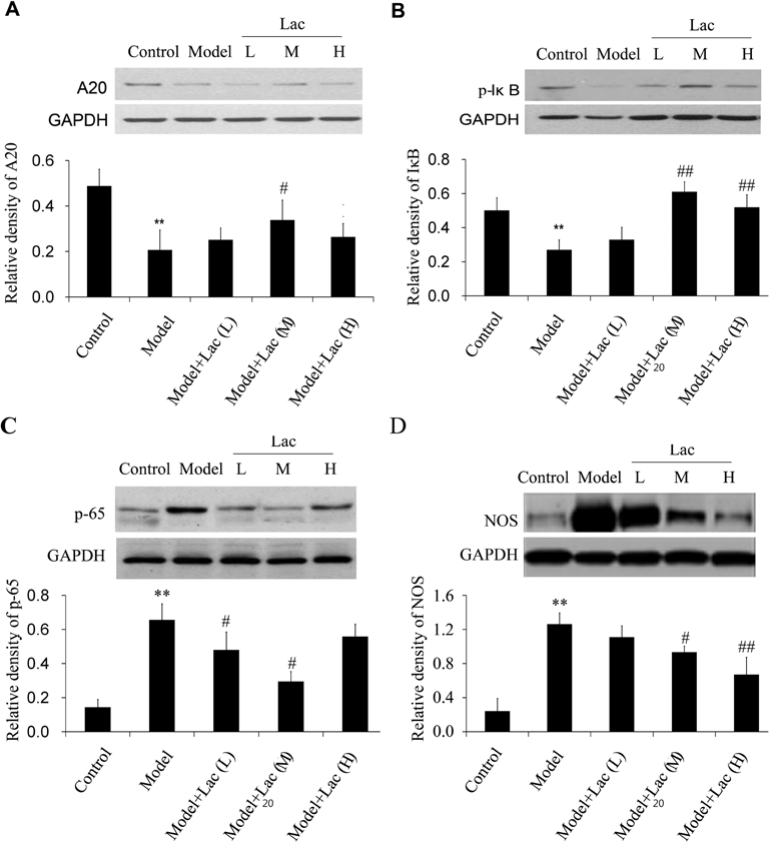
The expression levels of A20,p-IκB,p-65 NF-κB and NOS were assayed by Western blot (n=4) The expression levels of A20 (**A**), p-IκB (**B**), p-65 NF-κB (**C**), and NOS (**D**) were assayed by Western blot (*n*=4). The expression of A20 decreased, degradation of IκB increased, and NF-κB activation increased in model group. Lac can reverse the changes induced by MIRI. Besides, Lac weakened the expression of NOS. **P<0.01 compared with control; ^#^*P*<0.05 and ^##^*P*<0.01 compared with model group.

As shown in Figure 4A–C, the expression of A20 decreased, degradation of IκB increased and NF-κB activation increased obviously in model group (*P*<0.01) when compared with the control group. Moreover, the expression of NOS also decreased (Figure 4D) Inactivated Lac groups promoted the expression of A20 and IκB and inhibited the activation of p65, indicating that Lac inhibited the NF-κB signaling induced by MIRI.

NO is a reactive free radical which acts as an important regulator and biological mediator of numerous processes in cardiovascular systems. NO is formed from L-arginine by NO synthase (NOS). Next, we detected the expression of NOS in the ventricular tissues and found the expression of NOS was significantly increased in model group and Lac groups significantly weakened the expression of NOS.

## Discussion

Coronary heart disease (CHD) is currently more frequent in the elderly people with high disability and fatality rate, consuming a lot of medical resources for the prevention and treatment of the disease. Several studies have proved that its pathophysiological mechanism is MIRI [11].

Myocardial apoptosis is the important factor of intervention in the recovery of MIRI. The present study found that when MIRI occurs, myocardial endothelial cell and mitochondrial damage were aggravated and resulted into apoptosis; Lac can obviously improve the function of endothelial cells and relieve from endothelial cell injury and apoptosis. When MIRI occurs, the myocardial cell damage and the releases of myocardial enzymes such as CK and LDH increased, which are thought to be positive correlation with the degree of myocardial necrosis [12]. Therefore, the determination of serum myocardial enzyme activity can be used as one of the diagnostic criteria of MIRI damage degree. The results in the present study showed that Lac pretreatment can significantly inhibit the serum CK and LDH activities. When MIRI occurs, accumulation of ROS through various ways induces lipid peroxidation, leading to a series of pathophysiological changes such as damage to the structure of the biofilm, malfunction of ion transport, biomass production, and the function of the organelles, thereby aggravating ischemic myocardial tissue damage and dysfunction [13,14]. In addition to causing direct cell injury, ROS also increase leukocyte activation, chemotaxis, and leukocyte-endothelial adherence after MIRI. MDA, as the biological product of ROS and lipid peroxidation, reflects the degree of lipid peroxidation in the body and thus indirectly reflects the degree of myocardial injury. SOD is the main antioxidant enzymes on behalf of an organization’s ability to remove free radicals. The present study found that Lac increased serum SOD activity and decreased MDA content, further confirming the myocardial protection of Lac through inhibiting lipid peroxidation and increasing removal of ROS. Moreover, the presence of proinflammatory cytokines stimulates the NOS to synthesize large amount of NO, which may contribute to severe MIRI. The present study showed NOS correlated with injury probably through its proinflammatory role and Lac significantly weakened the expression of NOS.

A20, which is viewed as a potential therapeutic target for inflammatory disease is an NF-κB-inducible protein and negatively regulates inflammatory signaling pathways [15]. Lac can significantly decrease NF-κB expression and increase A20 expression of animals with MIRI, indicating that Lac is a hopeful prevention and therapeutic for MIRI. However, further study is still required to understand the detailed underlying mechanism of protective function of Lac in MIRI.

Our results also suggest that Lac pretreatment can inhibit lipid peroxidation and effectively improve the MIRI myocardial oxygen free radical damage through inhibiting NF-κB signaling, which has a wide range of applications in the prevention of ischemic diseases. However, there are several potential limitations in the current study. First, the *in vivo* side effects and mechanism of Lac are poorly determined. Further detailed study of Lac in anti-MIRI trials may be warranted. Second, although the obvious improvement against MIRI, the detailed use time and dosage need further study.

## Abbreviations

CK: creatine kinase
I–R: ischemia–reperfusion
Lac: *Lactobacillus*
LDH: lactate dehydrogenase
MDA: malondialdehyde
MIRI: myocardial I–R injury
NF: nuclear factor
NO: nitric oxide
NOS: NO synthase
ROS: reactive oxygen species
SOD: superoxide dismutase
